# miRLocator: Machine Learning-Based Prediction of Mature MicroRNAs within Plant Pre-miRNA Sequences

**DOI:** 10.1371/journal.pone.0142753

**Published:** 2015-11-11

**Authors:** Haibo Cui, Jingjing Zhai, Chuang Ma

**Affiliations:** 1 School of Computer Science and Information Engineering, Hubei University, Wuhan, 430070, China; 2 Center of Bioinformatics, College of Life Science, Northwest A&F University, Yangling, 712100, China; 3 Biomass Energy Center for Arid and Semi-Arid Lands, Northwest A&F University, Yangling, Shaanxi, 712100, China; Huazhong university of Science and Technology, CHINA

## Abstract

MicroRNAs (miRNAs) are a class of short, non-coding RNA that play regulatory roles in a wide variety of biological processes, such as plant growth and abiotic stress responses. Although several computational tools have been developed to identify primary miRNAs and precursor miRNAs (pre-miRNAs), very few provide the functionality of locating mature miRNAs within plant pre-miRNAs. This manuscript introduces a novel algorithm for predicting miRNAs named miRLocator, which isbased on machine learning techniques and sequence and structural features extracted from miRNA:miRNA* duplexes. To address the class imbalance problem (few real miRNAs and a large number of pseudo miRNAs), the prediction models in miRLocator were optimized by considering critical (and often ignored) factors that can markedly affect the prediction accuracy of mature miRNAs, including the machine learning algorithm and the ratio between training positive and negative samples. Ten-fold cross-validation on 5854 experimentally validated miRNAs from 19 plant species showed that miRLocator performed better than the state-of-art miRNA predictor miRdup in locating mature miRNAs within plant pre-miRNAs. miRLocator will aid researchers interested in discovering miRNAs from model and non-model plant species.

## Introduction

MicroRNAs (miRNAs) are small (~22 nucleotides), non-coding RNA molecules with important regulatory roles in gene expression. In plants, mature miRNAs are mostly generated from the cleavage of hairpin-structured miRNA precursors (pre-miRNAs) derived from long primary transcripts (pri-miRNAs)[1]. MiRNAs target a large number of protein-coding genes and are involved in various biological processes, including plant development, growth, abiotic stress responses and pathogen responses[2–4].The genome-wide identification of miRNAs is critical for obtaining a better understanding of the complex post-transcriptional regulation mechanisms involved in these biological processes. Next-generation sequencing (NGS) technologies have the ability to discover miRNAs in a high-throughput manner. However, this type of experimental method remains time-consuming because it requires the identification of expressed miRNAs from millions of sequencing reads and has a limited ability to detect miRNAs that exhibit low, linkage,stress, developmental and/or cell-specific expression[5]. Therefore, experimental technologies must be complemented with computational approaches to identify miRNAs at the genome scale, regardless of the availability of NGS sequencing data [5,6].

A number of computational approaches called miRNA predictors have been developed over the past few years, but most of these actually predict pri-miRNAs and/or pre-miRNAs [7]. The prediction of mature miRNAs involves determination of the location of mature miRNAs within pre-miRNA sequences. The existing computational approaches for miRNA prediction can be broadly separated into two categories: rule-based approaches[8,9] and machine learning (ML)-based approaches[5,6,9–13].

The rule-based approaches identify mature miRNAs based on several criteria determined through an analysis of known miRNAs, such as at most four unpaired nucleotides in putative miRNA duplexes and at least 70% sequence similarity with known miRNAs. This approach has inherent limitations for the identification of novel miRNAs with characteristics that differ from those of known miRNAs.

ML is a branch of artificial intelligence that applies various mathematical algorithms to improve the performance of prediction systems for the discovery of knowledge from novel datasets. ML was recently applied to the identification of mature miRNAs through the integration of thousands of sequence and structural properties extracted from miRNA sequences and/or miRNA duplexes. Notable ML-based miRNA predictors include miRdup[5], miRduplexSVM[6], miRAlign[9], MatureBayes[10], MaturePred[13] and ProMiR[14]. Although the application of ML technologies has resulted in a marked improvement in the miRNA prediction accuracy relative to that obtained with rule-based approaches, several problems in ML-based miRNA prediction remain unsolved.

First, the majority of these ML-based miRNA predictors were designed to identify animal miRNAs[9,10,12,14]. Therefore, only a few of these approaches can predict plant miRNAs[5,6,13], because the sequence and structural features of miRNA duplexes differ between animals and plants [1,5]. Second, all of these ML-based miRNA predictors (except miRdup) were developed based on a limited number of reference miRNAs compiled by their authors. Moreover, these predictors are no longer updated (re-trained) even when more experimentally validated miRNAs become available. In this study, we demonstrated that a larger sample size could provide a more accurate location of mature miRNAs within pre-miRNAs. Finally, the problem of class imbalance (a few positive samples [real miRNAs] and a large number of negative samples [miRNA-like segments]) were usually ignored in the prediction of mature miRNAs. It is unclear how the ratio between positive and negative samples (RPNS) affects the performance of ML-based miRNA predictors. Additionally, it is not known which RPNS is more suitable for unbalanced data.

In this manuscript, we present an ML-based system named miRLocator forthe computational prediction of mature miRNAs within plant pre-miRNAs. miRLocator was constructed based on 440 sequence and structural features extracted from miRNA duplexes. We explored the effect of class imbalance on the prediction performance of miRNA predictors and optimized the prediction models in miRLocator with an appropriate machine learning algorithm (random forest) and RPNS(1:5). A ten-fold cross-validation evaluation using 5854 experimentally validated miRNAs from 19 plant species demonstrated that the prediction performance of miRLocator was comparable to or better than that achieved with the state-of-art miRNA predictor miRdup. miRLocator was implemented in Python and provides functions for ML-based training and prediction processes using user-specified datasets. miRLocator is available at https://github.com/cma2015/miRLocator.

## Materials and Methods

### Flowchart for the Training and Testing of ML-Based miRNA Predictors

**Fig 1:**
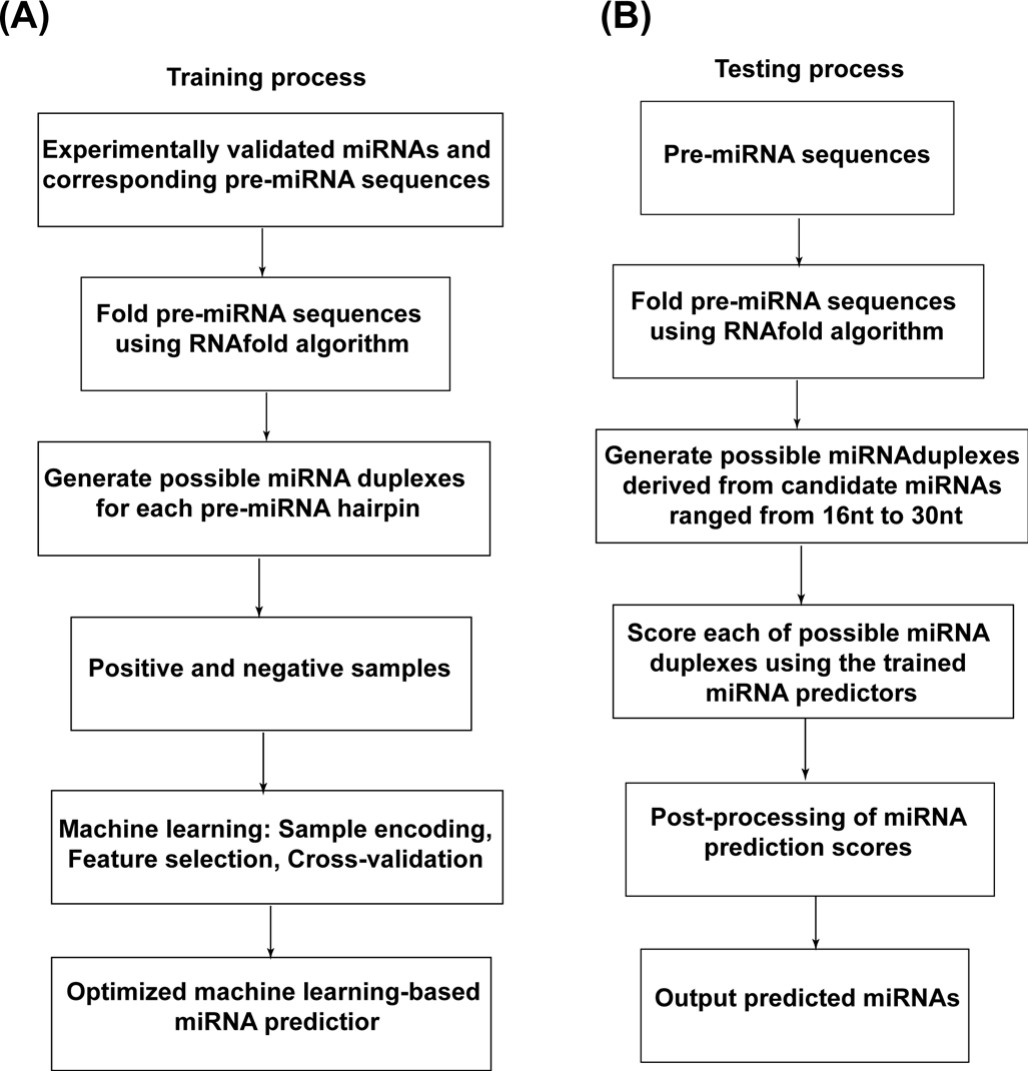
Training (A) and testing (B) procedures for ML-based miRNA prediction.

The general process for the training and testing of ML-based miRNA predictors is shown in Fig 1.

The training procedure involves the following steps (Fig 1A):

1. Obtain a set of experimentally validated miRNAs and their corresponding pre-miRNA sequences.
2. Fold the secondary structure of each pre-miRNA sequence using the RNAfold program in the ViennaRNA package (http://www.tbi.univie.ac.at/RNA; Version 2.1.8).
3. Generate positive and negative sample sets to train the ML-based miRNA predictors. miRLocator predicts mature miRNAs based on features extracted from miRNA duplexes. Therefore, the positive sample set should consist of miRNA duplexes derived from experimentally validated miRNAs. To avoid redundant information, only one miRNA duplex should be included in the positive sample set if both the 5' and 3' strands of the miRNA duplex are functional. The negative samples should consist of pseudo miRNA duplexes derived from segments randomly selected from pre-miRNA hairpins. In this study, the randomly selected segments were limited to the same sequence length as that of the experimentally validated miRNAs within the same pre-miRNAs. The ratio between positive and negative samples can be designated to obtain optimized prediction models.
4. Encode positive and negative samples with sequence and structural properties extracted from miRNA duplexes. The importance of each feature in distinguishing positive and negative samples can be measured through the random forest (RF)-based feature ranking algorithm (Gini importance) using the scikit-learn Python package (http://scikit-learn.org). The RF-based Gini importance calculates the mean decreases in impurity at each node of the trees obtained after the removal of the feature of interest from the feature matrix[15].
5. Build miRNA predictors using different ML algorithms and feature sets and evaluate the performance of miRNA predictors through the widely used 10-fold cross-validation algorithm. The best miRNA predictor can then be selected based on the experimental results from the 10-fold cross-validation.

The testing process was iteratively performed for each pre-miRNA. As shown in Fig 1B, the RNAfold algorithm was implemented first to obtain the secondary structure of the given pre-miRNA. All of the possible miRNA duplexes were then generated to predict candidate miRNAs ranging from 16nt to 30nt and scored using the trained miRNA predictor. A post-processing approach was subsequently applied to select the most probable miRNA as the prediction.

### Collection of Experimentally Validated miRNAs and Their Corresponding Pre-miRNAs

**Fig 2:**
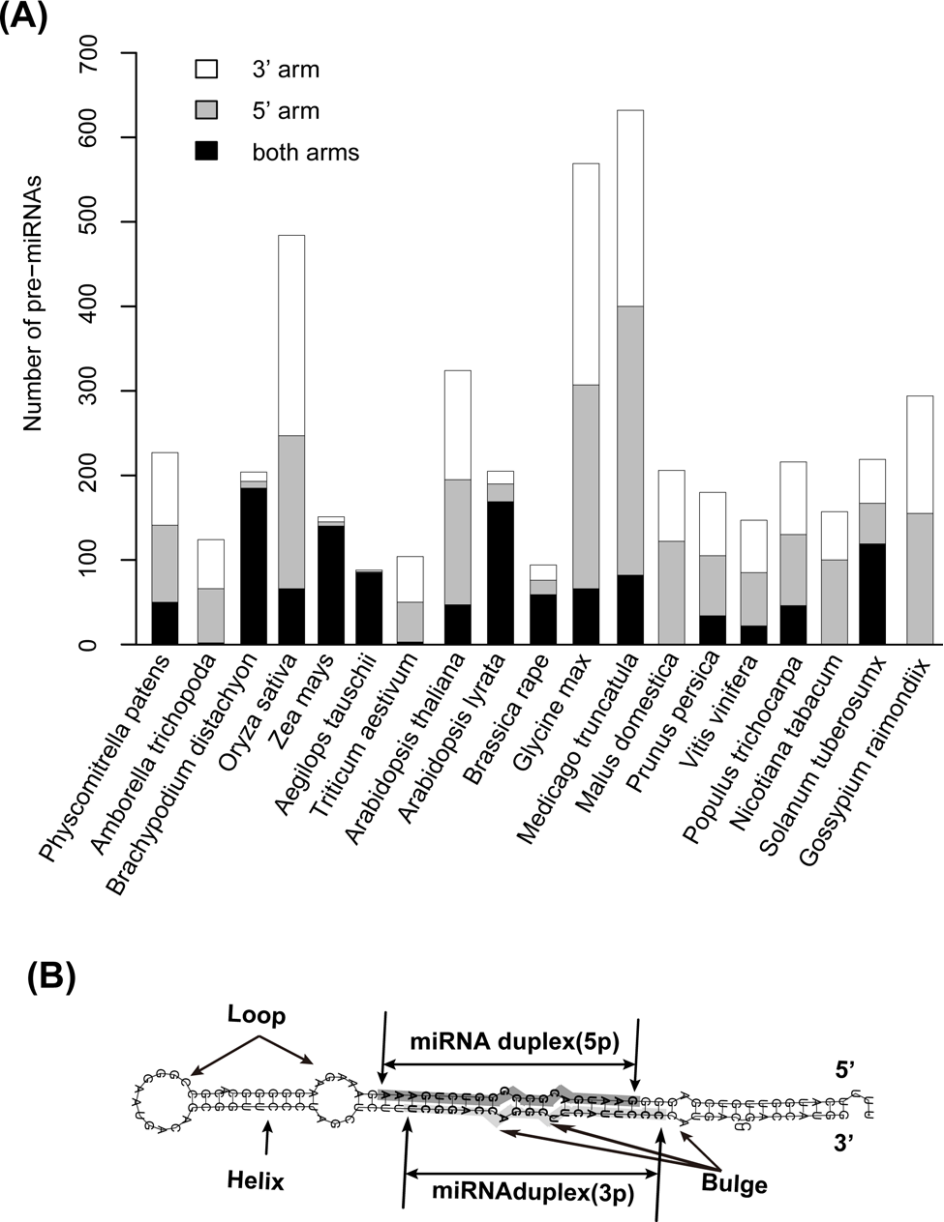
Experimentally validated miRNAs obtained from the miRBase database. (A) Statistical results of pre-miRNAs carrying experimentally validated miRNAs on their 5' and/or 3' arms. (B) Anatomy of the miRNA duplex in the pre-miRNA hairpin. "miRNA duplex (5p)" and "miRNA duplex (3p)" represent the 5' and 3' strands of the miRNA duplex, respectively. "Loop", "Helix" and "Bulge" are three common structural elements in the secondary structure of pre-miRNAs.

The miRNA data used in this study were obtained from the miRBase database (http://www.mirbase.org; Release 21), which is a collection of mature miRNAs and their corresponding pre-miRNAs generated through experimental and computational approaches. A total of 7009 experimentally verified plant miRNAs and their pre-miRNA sequences were automatically downloaded from the miRBase database using the miRdup software (http://www.cs.mcgill.ca/~blanchem/mirdup; Version 1.4) with the command "java -jar miRdup.jar -k Viridiplantae". To effectively evaluate the performance of miRNA predictors trained with miRNAs from individual species, we excluded 37 species annotated with less than 100 experimentally verified miRNAs. This filtration resulted in 5854 experimentally verified miRNAs generated from 4626 pre-miRNAs of 19 plant species (Fig 2 and S1 Table), including five monocotyledon plant species (*Brachypodiumdistachyon*, *Oryza sativa*, *Zea mays*, *Aegilopstauschii* and*Triticumaestivum*) and twelve dicotyledonous plant species within the *Brassicaceae*(*Arabidopsis thaliana*, *Arabidopsis lyrata*, and *Brassica rapa*), *Fabaceae*(*Glycine max* and *Medicagotruncatula*), *Rosaceae*(*Malusdomestica* and *Prunuspersica*), *Vitaceae* (*Vitisvinifera*), *Salicaceae*(*Populustrichocarpa*), *Solanaceae(Nicotianatabacum* and *Solanumtuberosum*) and *Malvaceae (Gossypiumraimondii*) families. Functional miRNAs generated from both the 5' and 3' arms of the pre-miRNAs were observed in almost all of the studied plant species with the exception of *M*.*domestica*, *N*.*tabacum* and *G*. *raimondii*(Fig 2).

### Feature Characterization of Mature miRNAs

Positive and negative samples were encoded with a numerical vector of length 440 that includedthe sequence and structural properties of the miRNA duplexes and their combinations(Table 1 and S2 Table).A total of 197sequence properties, including the miRNA sequence length, the types of nucleotides surrounding the start and end of the "miRNA duplex (5p)" and "miRNA duplex (3p)", and the mono- and di-nucleotide compositions in the miRNA sequence, were analyzed. In addition, 30 structural properties, including the number of base pairs, loops and bulges in the miRNA duplex and the minimum free energy (MFE) of the miRNA duplex, were selected based on previous studies related to miRNA prediction [5]. We also introduced 22 structural features related to the positional entropy of the RNA secondary structure. Positional entropy can be used to quantify the ‘cleavage specificity’ of each position in an RNA fragment [16] and was defined using the following formula:

$$\text{H}(\text{i})\;=\;{\text{log}}_{2}(-\sum_{\text{j}}({\text{p}}_{\text{i},\text{j}}{\text{logp}}_{\text{i},\text{j}}+(1-{\text{p}}_{\text{i},\text{j}})\text{log}(1-{\text{p}}_{\text{i},\text{j}}))),$$ (1)

where 1 ≤ *i* < *j* ≤ *n*, *n* is the pre-miRNA sequence length and *p*_*ij*_ represents the probability of base pairing between *i* and *j*. *H(i)* ranged from 0 (*p*_*ij*_ = 1 for some *j*) to log_2_(*n*) (*p*_*ij*_ = 1/*n* for each *j*).

**Table 1:** Sequence and structural features used in miRLocator.

Type	Feature Number	Feature Description
miRNALen	1	miRNA length
MNC	4	Frequency of four nucleotides
DNC	16	Frequency of 16 dinucleotides
NT(5p)	(5+1+5)*4*2	Type of nucleotides surrounding the start and end of the miRNA duplex (5p) within the [-5,5] region. A, C, G and U are encoded as0001, 0010, 0100, and 1000, respectively.
NT(3p)	(5+1+5)*4*2	Type of nucleotides surrounding the start and end of the miRNA duplex(3p) within the [-5,5] region.
MFE	1	Minimum free energy (MFE) of the miRNA duplex
mlBulge	1	Maximal length of an miRNA without bulge in the miRNA duplex
bpNum	1	Number of base pairs in the miRNA duplex
dist2 Loop	1	Distance of the duplex to the terminal loop
dist2Helix	1	Distance of the duplex to the start of the helix
numLoop	1	Number of loops in the miRNA duplex
numBulges	1	Number of bulges in the miRNA duplex
perfectBP	3*2	Presence and start position of perfect base pairs with 5, 10, and 20nt
numBP_Win*X*	3	Average number of base pairs obtained by scanning the miRNA duplex with a window of length *X*nt (*X* = 4, 6, and 8)
Bulges	7*2	Presence of bulges in the area surrounding the start and end of the miRNA within the [-3,3] region
posEntropy	(5+1+5)*2	Entropy values of the areas surrounding the start and end of the miRNA in the[-5,5] region
monoSSq	4*3+1	Number of different combinations of four nucleotides (A,C,G, and U) and three structure symbols (‘(‘, ‘)’, and ‘.’). One additional feature was added for undefined combinations. ‘(‘, ‘)’, and ‘.’ represent paired, paired, and unpaired nucleotides in the secondary structure of the pre-miRNA, respectively.
diSS	16*9+1	Number of different combinations of 16 dinucleotides and nine di-structure symbols. One additional feature was added for undefined combinations.
triplets	32+1	Number of 32 combinations of mononucleotide and triplets of structure symbols. One additional feature was added for undefined combinations.

The remaining 191 features consisted of different combinations of the sequence and structural properties extracted from the miRNA duplexes (‘monoSSq’, ‘diSS’, and ‘triplets’). For example, ‘A(‘ in the ‘monoSSq’ list represents an A nucleotide base-paired in the miRNA duplex, ‘AU(.’ in the ‘diSS’ list indicates a paired A nucleotide with a neighboring unpaired U nucleotide in the miRNA duplex, and ‘A(.)’in the ‘triplets’ list represents an unpaired A with two paired neighboring nucleotides in the miRNA duplex.

### Machine Learning Algorithms

Many ML algorithms have been developed to construct binary classifiers in the fields of bioinformatics and computational biology [17].In this study, we focused on five widely used ML algorithms: random forest (RF), support vector machine (SVM), naïve Bayesian network (NB), decision tree (DT) and k-nearest neighbors (kNN). The RF algorithm involves an ensemble of decision trees grown based a randomly selected subset of samples and features[18]. SVM maps input data into a high-dimensional feature space with a linear or non-linear kernel function (i.e., radial basis function [RBF]) and formulates the classification problem to find the optimal hyper-plane with the minimal expected classification error for the test samples [14]. The optimal hyper-plane can be determined with the penalty parameter *C* and various kernel function parameters, such as gamma (*γ*) for the RBF kernel. The NB is another supervised ML algorithm based on the application of Bayes' theorem, which assumes that the likelihood of the features is Gaussian. The kNN is a type of instance-based ML algorithm that classified a sample based on the majority of the k-nearest neighbors. All of these ML algorithms were implemented with functions in the "scikit" library written in the Python programming language.

### Cross-Validation Algorithm

Cross-validation is a commonly used approach to evaluate the binary prediction performance of ML systems. For the ten-fold cross-validation algorithm, positive and negative samples are randomly divided into ten subsets of approximately equal sizes. For each round of cross-validation, nine of these ten subsets are selected to form the training datasets used to train the ML systems, and the tenth subset is used as the testing dataset to evaluate the prediction performance of the trained ML system throughreceiver operating characteristic (ROC) curve analysis. The ROC curve reflects the variability of the true positive rate (sensitivity) against the false positive rate (1-specificity) at all possible thresholds. The value of the area under the curve (AUC) can range from 0 to 1, with a higher AUC value indicating that the classifier has a higher discriminative capability to differentiate positive samples from negative samples. After all of the subsets are used as the testing dataset, the mean value of the ten AUCs is considered the final performance of ML systems.

### Post-Processing of Prediction Scores to Locate Mature miRNAs within Pre-miRNAs

Because the location and length of the mature miRNAs are unseen by the miRNA predictors during the testing procedure, all of the possible miRNA duplexes with candidate miRNAs ranging from 16nt to 30nt were generated for each tested pre-miRNA sequence and scored using the trained miRNA predictor. We applied a post-processing approach to determine the length and location of the predicted miRNAs by considering the score of all of the candidate miRNAs. For a given position *p* on the pre-miRNA hairpin, the summed prediction score was calculated as

$$\text{S}(\text{p})\;=\;\sum_{16\leq \text{L}\leq 30}\;\text{score}(\text{p},\text{L}).$$ (2)

The start position of the predicted miRNA(*p*_*pred*_) was identified as the position at which *S(p)* reaches the maximal value. The length of the predicted miRNA was determined as the length that yields the maximal value of *score(p*_*pred*_, *L)*. miRLocator outputs the predicted miRNA and its corresponding passenger strand for the tested pre-miRNA sequence.

The accuracy of different ML prediction systems in predicting mature miRNAs within pre-miRNAs was evaluated by comparing the start and end positions of the predicted miRNAs with those of the true miRNAs[5].The cumulative frequency of the correctly predicted miRNAs was calculated at different resolutions *d*(0 ≤ *d* ≤ 10nt). In this manuscript, the resolution *d* represents the start (end) of the predicted miRNAs located within *d* nucleotides of the true start (end) position.

## Results and Discussion

### Performance Evaluation of miRNA Predictors Constructed with Different ML Algorithms

In the binary classification case, appropriate ML algorithms are required to address the sample imbalance problem. Following the training procedure shown in Fig 1A, we evaluated the performance of different ML algorithms for the classification of real and pseudo miRNA duplexes. First, we generated a positive sample set consisting of 4505 miRNA duplexes supported with experimentally validated miRNAs and a negative sample set consisting of approximately 4505*N (*N* = 1, 5, 10 and 50) pseudo miRNA duplexes derived from randomly selected segments within the pre-miRNA hairpins. We then encoded each sample with 440 numeric features (Table 1 and S2 Table) and performed ten-fold cross-validation and ROC analyses to evaluate the performance of the miRNA predictors constructed with five ML algorithms(RF, SVM, NB, kNN and DT).

**Fig 3:**
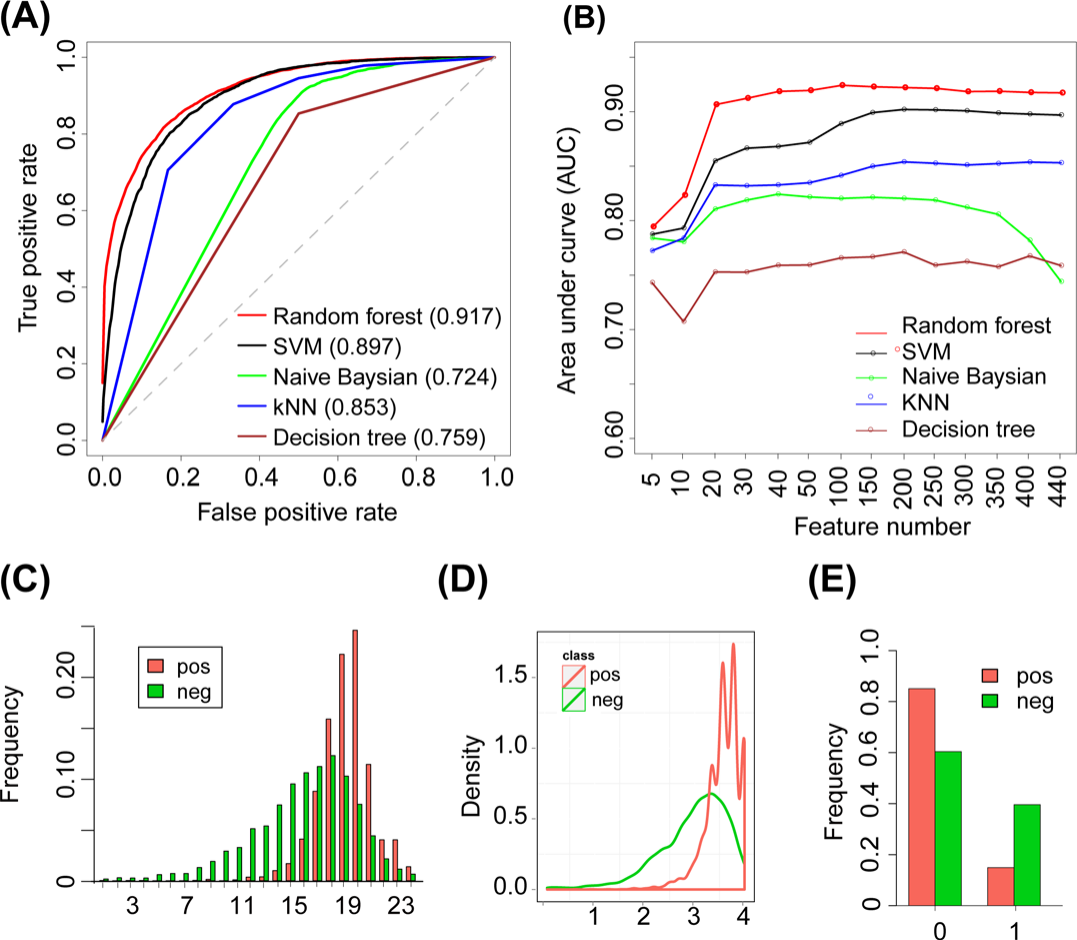
Performance of ML-based miRNA predictors in classifying real and pseudo miRNA duplexes. (A)ROC curve displaying the performance of different ML-based miRNA predictors in the ten-fold cross-validation experiment.(B) Performance of ML-based miRNA predictors obtained with different numbers of features.(C) Distribution of the number of base pairs in the positive and negative sample sets.(D) Distribution of the average number of base pairs in a 4-nt sliding window in the positive and negative sample sets.(E) Frequency of bulges 1nt upstream of the miRNA end in the positive and negative sample sets.

At an RPNS of 1:1, the AUC (area under the ROC curve) values for RF, SVM, NB, kNN and DT were 0.917, 0.897, 0.724, 0.853 and 0.759, respectively (Table 2, Fig 3A). Comparable AUC values were obtained for each of the tested ML algorithm at RPNS values of 1:5, 1:10 and 1:50 (Table 2). These results indicate that the performance of the different ML algorithms in classifying positive and negative samples is only slightly affected by the RPNS. This finding was likely mainly obtained because the length of the pseudo miRNAs in the negative sample set were limited that of the real miRNAs within the same pre-miRNA sequences.

**Table 2:** AUC values of miRNA predictors constructed with different ML algorithms using different RPNSs.

Ratio	RF	SVM	NB	kNN	DT
1:1	0.917	0.897	0.724	0.853	0.759
1:5	0.930	0.888	0.725	0.861	0.752
1:10	0.938	0.878	0.723	0.851	0.738
1:50	0.938	0.876	0.727	0.848	0.728

The contribution of each feature to the classification of positive and negative samples was measured using the RF-based Gini importance algorithm (see Materials and Methods section). At an RPNS of1:1, the top five features were in the order bpNum> numBP_Win4 > numBP_Win6 > BulgeAtEnd_u1 > numBP_Win8 (Table 3 and S2 Table). The importance of these features was also demonstrated by the differences in their distributions among the positive and negative samples (Fig 3C–3E and S1 Fig). The analysis of the bpNum feature revealed that more than 83% of the positive samples contained at least 18 base pairs in the duplexes, whereas this proportion was lower than 40% in the negative sample set (Fig 3C). For the numBP_Win4feature, 94.6% of the positive samples had a score of at least 3, whereas the corresponding proportion in the negative sample set was only 58.4% (Fig 3D). The evaluation of the BulgeAtEnd_u1feature showed that only 14.9% of the positive samples had a bulge 1nt upstream of the miRNA end, whereas the respective proportion in the negative sample set was 38.6% (Fig 3E).Notably, our 22 introduced positional entropy-related features also contributed to the identification of the miRNA duplexes, with eight of the features belonging to the top50 features (S2 Table).

**Table 3:** Feature importance scores evaluated with the RF-based Gini importance algorithm.

Features	Description	1:1^a^	1:5 ^a^	1:10 ^a^	1:50 ^a^
bpNum	Number of base pairs in the duplex	0.020 [1]	0.012[3]	0.009[3]	0.006[11]
numbP_Win4	Average number of base pairs in a sliding window of 4nt	0.020[2]	0.015[1]	0.010[1]	0.006[4]
numBP_Win6	Average number of base pairs in a sliding window of 6nt	0.018[3]	0.013[2]	0.010[2]	0.006[6]
BulgeAtEnd_u1	Bulge located 1nt upstream of the miRNA end	0.016[4]	0.008[6]	0.006[14]	0.003[163]
numBP_Win8	Average number of base pairs in a sliding window of 8nt	0.014[5]	0.010 [5]	0.008[5]	0.005[16]
BulgeAtEnd_u2	Bulge located 2nt upstream of the miRNA end	0.012[6]	0.007[14]	0.005[31]	0.003[177]
BulgeAtEnd_u3	Bulge located 3nt upstream of the miRNA end	0.011[7]	0.006[19]	0.005[40]	0.003[111]
Persent_10mer	Presence and start position of perfect base pairs in a length of 10nt	0.011[8]	0.008[7]	0.006[13]	0.003[103]
U)	Paired U nucleotide in the miRNA duplex	0.010 [9	0.006[17]	0.005[25]	0.003 [113]
mlBulge	Maximal miRNA length without a bulge in the duplex	0.009[10]	0.007 [12]	0.007[10]	0.005 [26]

^a^The rank of each feature is presented in the bracket.

Feature ranking was repeated for different RPNSs. Some features, such as bpNum, mlBulge and numbp_Win4,6, and 8, were ranked at the top with different RPNSs, whereas other features, such as BulgeAtEnd_u1, Persent_10mer, and U), were ranked differently with different RPNSs (Table 3 and S2 Table). Because the contributions of some features were significantly affected by the RPNSs, we preferred to include all 440 features in the prediction of mature miRNAs. However, the top 100 informative features can be used to obtain effective predictions usingRF and SVM (Fig 3B).

Overall, RF and SVM are two of the most powerful ML algorithms for the identification of mature miRNAs. Considering the computational time required for optimizing parameters in SVM, the RF algorithm and all 440 features were applied in miRLocator to construct the prediction model formature miRNAs.

### Performance Evaluation of miRNA Predictors in Predicting Mature miRNAs within Plant Pre-miRNAs

We then evaluated the performance of miRLocator and previous miRNA predictors in locating mature miRNAs within pre-miRNA sequences from plants. A given set of pre-miRNAs was divided into ten subsets, nine of which were selected to constructpositive and negative sample sets for trainingthe miRNA predictors, whereas the tenth subset was used to evaluate the performance of the trained miRNA predictors. The prediction accuracy of the miRNA predictors was calculated by comparing the start (end) positions of the predicted miRNAs with the start (end) of the true miRNAs.

To ensure a fair comparison, different miRNA predictors should be trained with the same training dataset and assessed with the same testing dataset. However, to the best of our knowledge, only the recently published miRNA predictor miRdup provides functions to implement the ML-based training and testing procedures using user-provided datasets. By combining lineage-specific features with the RF algorithm, miRdup achieves a superior prediction accuracy relative to that obtained with other miRNA predictors, including MatureBayes, miRAlign, MaturePred and ProMiR[5].

**Fig 4:**
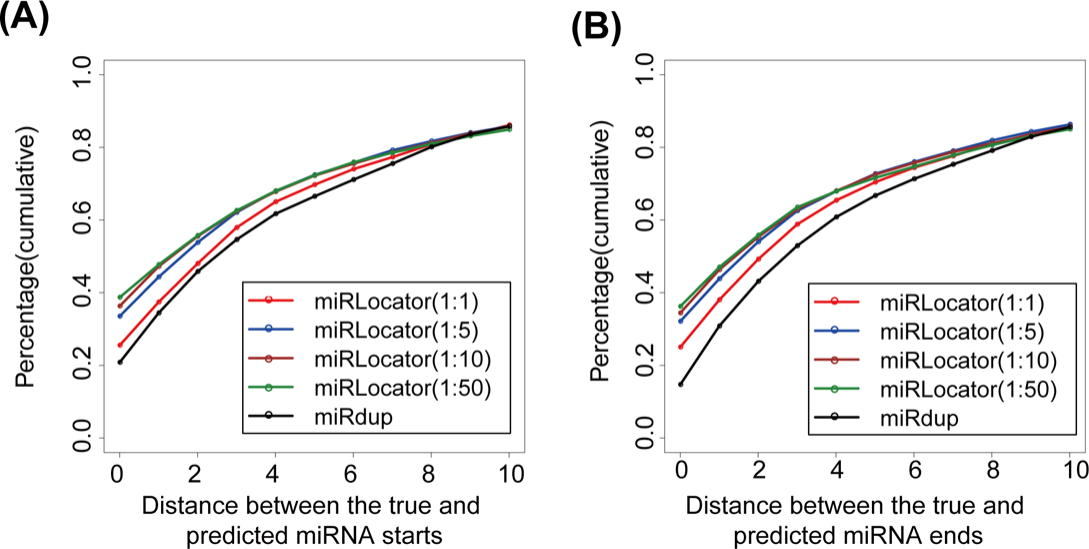
Cumulative frequency of correctly predicted start (A) and end (B) positions of miRNAs at different resolutions. For a given resolution *d* (0 ≤ *d* ≤ 10 nt), the start (end) of the predicted miRNAs was regarded as true if it was located within *d*bp from the start (end) of the annotated miRNAs.

Fig 4 shows the cumulative frequency of the miRNA start and end positions that were correctly predicted by miRdup and miRLocator. At an RPNS of 1:1, miRLocator perfectly predicted (*d* = 0) the start and end positions for 25.5% and 25.0% of the true positions, respectively. In contrast, miRdup made perfect predictions of start and end positions in 20.8% and 14.7% of the cases, respectively. The prediction performance of miRLocator was superior or comparable to that of miRdup at all of the other resolutions analyzed. There are two possible reasons for this result. First, miRLocator characterized miRNAs with more sequence and structural features, some of which (21 positional entropy-related features) were applied for the first time to the prediction of mature miRNAs. Second, miRLocator applied an improved post-processing approach to locate mature miRNAs within the pre-miRNAs by considering the prediction score of all possible miRNA duplexes. In contrast, miRdup focused on the possible miRNA duplexes with prediction scores higher than 0.99, leading to the failure to accurately locate the start and end positions of the mature miRNAs within a proportion of pre-miRNA sequences [6].

We further explored whether the performance of miRLocator could be affected by the RPNS. As the RPNS was decreased from 1:1 to 1:5, the perfect prediction of the start positions (*d* = 0) produced by miRLocator increased from 25.5% to 33.5%, and the perfect prediction of the end positions obtained by miRLocator increased from 25.0% to 32.1% (Fig 4). The use of more negative training samples slightly improved the performance of miRLocator (Fig 4). These results demonstrate the importance of RPNS in the accurate prediction of mature miRNAs. To avoid biased prediction models with a preference for pseudo miRNAs, an RPNS of 1:5 should be used in the development of ML-based miRNA predictors.

### Effect of the Sample Size on the Performance of miRNA Predictors

In previous studies, researchers typically constructed ML-based miRNA predictors using all plant miRNAs to increase the number of positive training samples. However, the effect of the positive sample size on the performance of miRNA predictors has not yet been investigated. Therefore, the number of miRNAs required to efficiently construct miRNA predictors is unknown. Additionally, it remains unclear whether the prediction accuracy of mature miRNAs is acceptable or whether miRNA predictors should be trained with a limited number of miRNAs from individual plant species.

**Fig 5:**
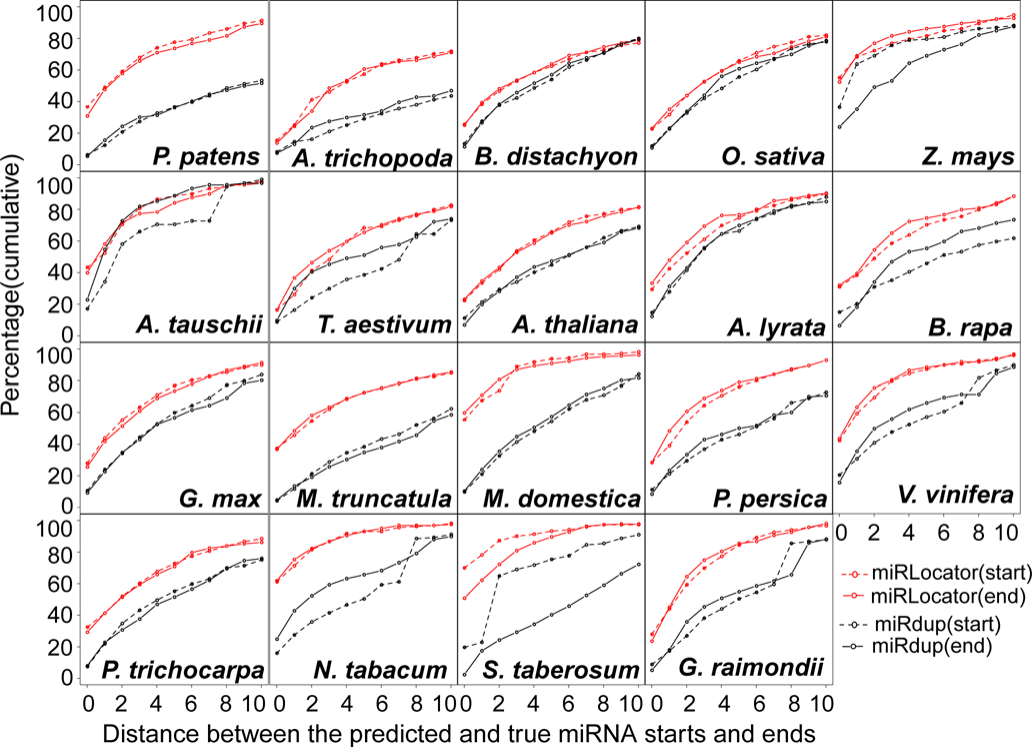
Effect of sample size on the prediction accuracy of miRLocator and miRdup.

Based on the experimentally validated miRNAs collected from19 plant species, we observed that the distributions of some miRNA-related features differed between species. For instance, the miRNAs from *A*. *thaliana* were generally 21-nt in length and were almost never longer than 23nt, whereas the miRNAs from *M*. *truncatula* had a broader length range (20nt ~ 24nt) (S2 Fig).To eliminate the effect of species specificity, an evaluation experiment was performed using 715 experimentally validated miRNAs from *M*. *truncatula*. We first randomly selected a set of experimentally validated miRNAs (*N* = 50, 100, 150, 200, 250, 300, 350, 400, 450, 500, 550, 600, and 650) and their corresponding pre-miRNA sequences. We then evaluated the performances of miRLocator and miRdup with these selected miRNAs and calculated the cumulative frequency of the correctly predicted start and end positions of the miRNAs at different resolutions using miRLocator and miRdup. This process was repeated 20 times. We subsequently drew abox plot of the cumulative frequencies at are solution of *d* = 5 for the different sample sizes. With increases in the sample size, the prediction accuracy of miRLocator increased from 0.61 to 0.73 and became relatively stable once the number of positive training samples reached 400 (Fig 5). In contrast, the prediction accuracy of miRdup gradually increased from 0.32 to 0.62at the number of positive training samples was increased from 50 to 650 (Fig 5). These results indicated that (1) miRdup strongly depends on the training sample size to effectively predict mature miRNAs and (2) miRLocator is an option if the number of experimentally validated miRNAs in the training dataset is limited (<400).

**Fig 6:**
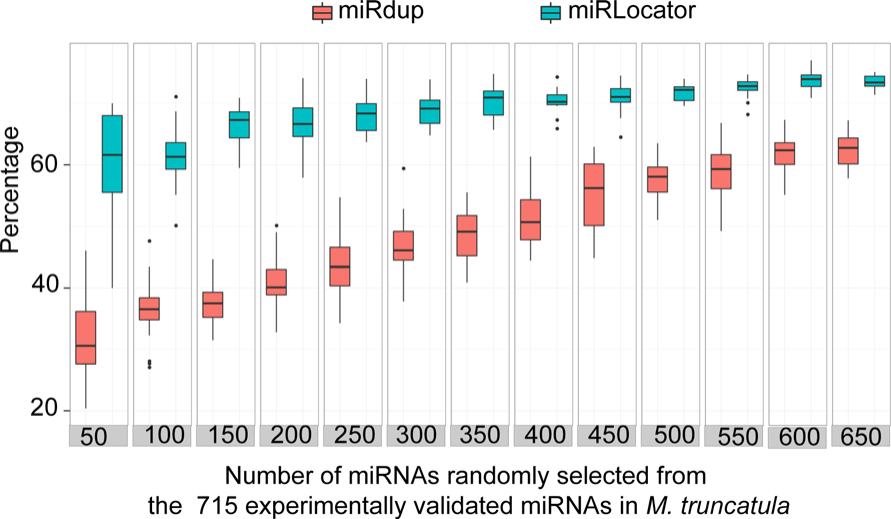
Cumulative frequency of correctly predicted start and end positions of miRNAs at different resolutions.

To confirm these results, we assessed the performances of miRdup and miRLocator with a limited number of experimentally validated miRNAs from individual species. As expected, the prediction performance of miRLocator was comparable to or better than that of miRdup in all tested situations (Fig 6).

### Implementation of miRLocator

miRLocator was implemented as a Python program running on a Linux/Unix platform that requires pre-installation of the ViennaRNA package (http://www.tbi.univie.ac.at/RNA), Python (v2.7.6) programming language and Python-related libraries, including sklearn (v0.16.1; http://scikit-learn.org), NumPy (v1.9.1; http://www.numpy.org) and SciPy (v0.15.0; http://www.scipy.org). The source codes of miRLocatorcan be downloaded from https://github.com/cma2015/miRLocator. The RF-based miRNA prediction models in miRLocator were implemented using the "RandomForestClassifier" function in the "sklearn" library and can be automatically trained with user-specified miRNA datasets. For a given pre-miRNA sequence, miRLocator first scores all of the miRNA candidates with sequence lengths ranging from 16nt to 30nt and then outputs the most probable miRNA and its corresponding passenger strand as the final prediction result.

## Conclusion

A new computational approach (miRLocator) to predict mature miRNAs within plant pre-miRNAs was developed. We then demonstrated the importance of the ML algorithm, the sample size and the ratio between training positive and negative samples (RPNS) to address the class imbalance problem observed in the development of ML-based miRNA predictors with high prediction performance. We also determined the appropriate ML algorithm (RF) and RPNS (1:5) for the prediction of mature miRNAs. miRLocator can be used to more accurately locate mature miRNAs within plant pre-miRNA sequences compared with the state-of-art miRNA predictor miRdup. Combined with pre-miRNA predictors[19,20], miRLocator would aid the identification of miRNAs from model and non-model plant species.

## Supporting Information

S1 Fig Distribution of numbp_Win4 (A) and numBP_Win8 (B) in the positive and negative sample sets. Numbp_Win4 and numbp_Win8 denote the average number of base pairs in a sliding window of 6nt(a) and 8nt(b), respectively.
(TIF)

S2 Fig Distribution of miRNA lengths in *A*. *thaliana* (A) and *M*. *truncatula*(B). (TIF)

S1 Table The 5854 experimentally validated miRNAs used in this study. (XLSX)

S2 Table The 440 features used to characterize the miRNA duplexes. (XLS)
