# Supplementary figures and images for: miRLocator: Machine Learning-Based Prediction of Mature MicroRNAs within Plant Pre-miRNA Sequences
Source: PLoS One. 2015 Nov 11;10(11):e0142753. doi: 10.1371/journal.pone.0142753 (PMC4641693; doi:10.1371/journal.pone.0142753)

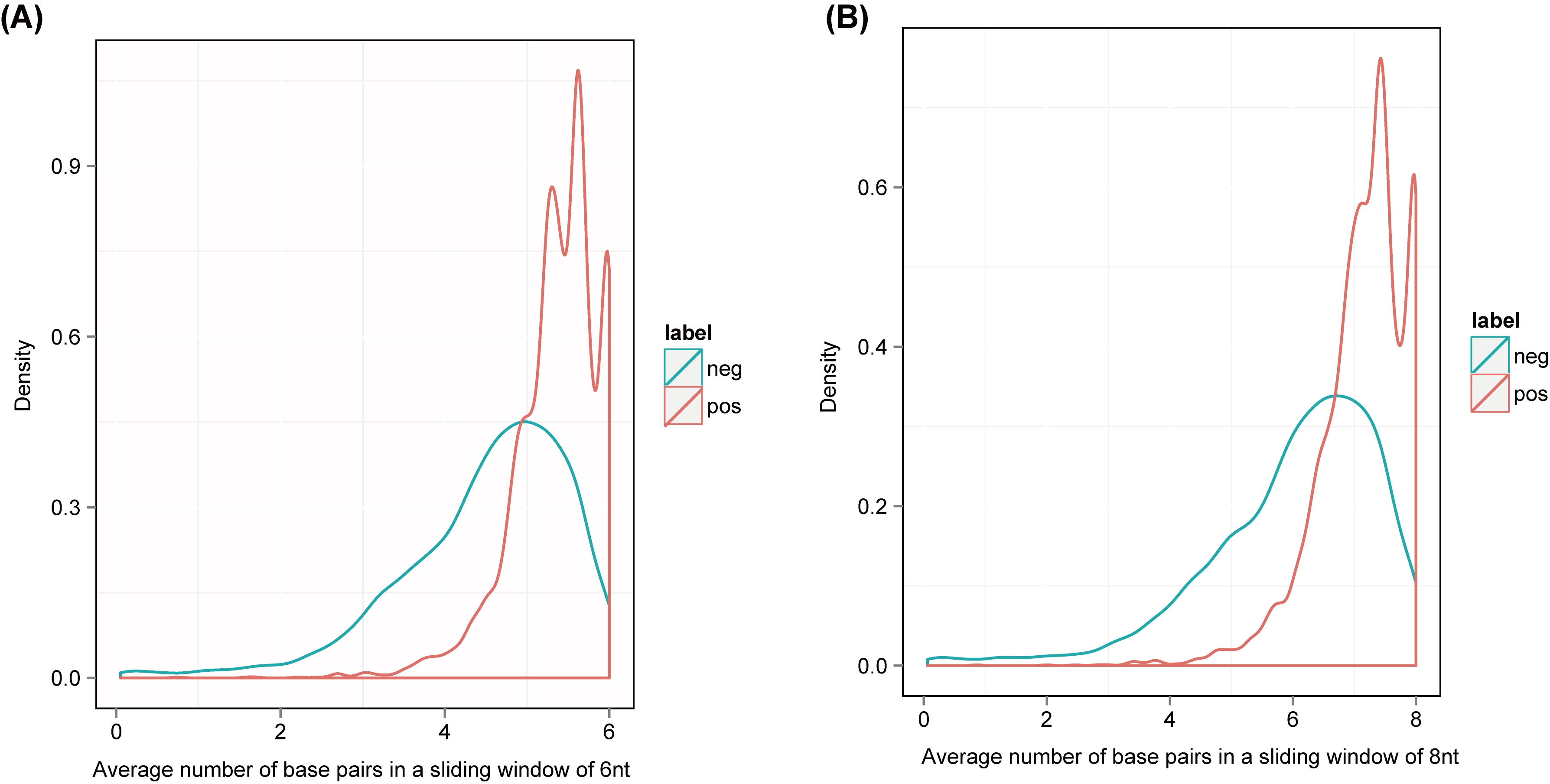

Supplement: S1 Fig — Numbp_Win4 and numbp_Win8 denote the average number of base pairs in a sliding window of 6nt(a) and 8nt(b), respectively. (TIF) [file pone.0142753.s001.tif]

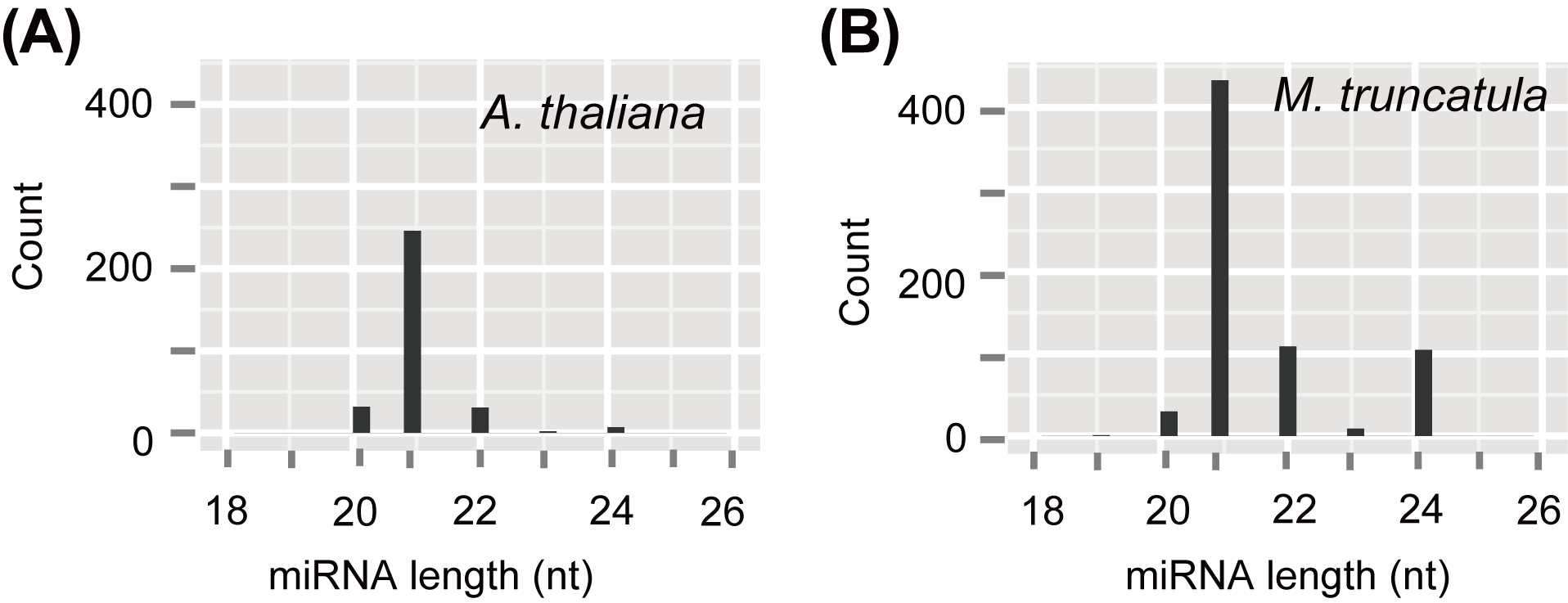

Supplement: S2 Fig — (TIF) [file pone.0142753.s002.tif]
